# Is ultrasonic investigation of transverse tracheal air shadow diameter reasonable for evaluation of difficult airway in pregnant women: A prospective comparative study

**DOI:** 10.12669/pjms.301.3972

**Published:** 2014

**Authors:** Meltem Turkay Aydogmus, Kerem Erkalp, Sitki Nadir Sinikoglu, Taner A. Usta, Gunes O. Ulger, Aysin Alagol

**Affiliations:** 1 Dr. Meltem Turkay Aydogmus, Department of Anesthesiology and Reanimation, Bagcilar Training and Research Hospital, Turkey.; 2 Dr. Kerem Erkalp, Department of Anesthesiology and Reanimation, Bagcilar Training and Research Hospital, Turkey.; 3 Dr. Sitki Nadir Sinikoglu, Department of Anesthesiology and Reanimation, Bagcilar Training and Research Hospital, Turkey.; 4 Dr. Taner A. Usta, Department of Obstetrics and Gynaecology, Bagcilar Training and Research Hospital, Turkey.; 5 Dr. Gunes O. Ulger, Department of Anesthesiology and Reanimation, Bagcilar Training and Research Hospital, Turkey.; 6 Research Assistant,; 7 Dr. Aysin Alagol, Department of Anesthesiology and Reanimation, Bagcilar Training and Research Hospital, Turkey.

**Keywords:** Pregnancy, body mass index, obesity, airway evaluation, ultrasonography, diameter of transverse tracheal air shadow

## Abstract

***Objective:*** The aim of this study was to compare clinical screening tests (modified Mallampati score, Cormack-Lehane score, thyromental distance, and sternomental distance) with ultrasonic measurements of the upper airway in predicting difficult intubation in pregnant women whose Body Mass Index (BMI) is higher and lower than 30 kg m-2.

***Methods:*** This study was designed as a prospective observational trial, and consisted of 40 pregnant women of American Society of Anesthesiologists (ASA) 1-2 groups. Patients with a BMI lower than 30 kg m-2 were included in Group 1 (n=20), and patients with a BMI higher than 30 kg m-2 were included in Group 2 (n=20). In the supine position with head in mild extension, the diameter of the transverse tracheal air shadow in the subglottic area of the front neck was measured using ultrasonography. Modified Mallampati score, Cormack-Lehane score, thyromental distance and sternomental distance measurements were recorded.

***Results:*** No statistically significant difference was detected between groups regarding mean age, mean number of pregnancy, ASA scores and comorbid disease. Mean body weight (p=0.0001) and mean pre-pregnancy weight (p=0.0001) were significantly higher in Group 2. There was no statistically significant difference between groups regarding mean modified Mallampati score, thyromental distance, sternomental distance measurements, Cormack-Lehane score, and mean ultrasonic measurements.

***Conclusion:*** It was found that BMI higher or lower than 30 kg m-2 has no effect on ultrasonic measurements and clinical airway tests. We thought that ultrasonic measurement could not give us valuable information in obese or non-obese pregnant women.

## INTRODUCTION

Anesthesia causes deaths directly effect maternal mortality and its airway-related factors.^1^ Airway management in obstetrics remains an important problem for several reasons.^2^ In the United States of America anesthesia is the seventh most common cause of maternal death. Changes in airway anatomy and physiology during pregnancy can lead to airway management problems.^3^ During pregnancy, the diameter of the laryngopharyngeal tract can tighten, and the modified Mallampati score could worsen.^4^ Thus, hypoxia may occur faster. Additional morbidities such as obesity and preeclampsia can cause problems in airway management. The incidence rate of difficult intubation in pregnancy is 1 per 30 births, according to the literature. The incidence rate of failed intubation in pregnancy is 1 per 280 births, which is 8 times more than that of the normal population. It is better to evaluate several criteria in understanding difficult intubation.^2^

Although the role of ultrasonography (USG) in anesthesia-related airway evaluation and procedural inventions is not clear, its use should be supported.^5^ Anatomical structures in supraglottic, glottic, and subglottic areas can be monitored using USG. In airway management, USG can be useful in confirming endotracheal tube placement, in suggesting post-extubation stridor, in the evaluation of soft mass tissues before intubation, and in the determination of pediatric endotracheal tube size.^6^ Airway monitoring via USG has several advantages, including the fact that it is reliable, fast, repeatable and transportable.^7^

Changes in the respiratory system during pregnancy are manifest as alterations in the upper airway, also minute ventilation, lung volumes and arterial oxygenation. The pre-operative anaesthesia evaluation should include an assessment of airway.^8^ It has been reported that difficult intubation increases with increasing Body Mass Index (BMI).^9^ The tracheas of obese patients are believed to be more difficult to intubate than those of normal weight patients.^10^ There are lots of factors associated with difficult laryngoscopy which include short sternomental distance, short thyromental distance, large neck circumference, limited head, neck and jaw movement, receding mandible and prominent teeth. But, there are a few simple pre-operative bedside determinations that can be performed quickly to evaluate the airway in a pregnant patient. These include mouth opening, Mallampati classification, thyromental distance and atlanto-occipital extension. *It is recommended that the airway be reassessed before induction of general anaesthesia.*^11^ Soft tissue changes such as airway edema are an invariable association of pregnancy, and this may contribute to difficult intubation. The underlying cause for this change is attributed to fluid retention that occurs with pregnancy. Upper airway volume has two components: an oral component and a pharyngeal component. The oral component is the one normally assessed using Mallampati classification. The pharyngeal volume is concealed and not evaluated in clinical practice.^4^

The aim of this study was to compare the ultrasonic measurements of the transverse diameter of the upper airway and clinical scanning tests for difficult intubation in pregnant women with a BMI higher and lower than 30 kg m^-2^. Is USG useful or not to evaluate the subglottic area volume changes in pregnant patients according to BMI?

## METHODS

This study was a prospective observational trial and conducted in Bagcilar Training and Research Hospital. Permission was granted by the hospital’s ethics committee, and written consent was obtained from patients. Forty pregnant women of American Society of Anesthesiologists (ASA) 1-2 scheduled for general anesthesia for cesarian operation were included in the study, which took 4 months to be completed. Patients with airway malformation and pathology causing limitations in head-neck movements were excluded. Diabetes mellitus and chronic hypertension were evaluated as comorbid diseases.

The degree of obesity was calculated according to the classification of the BMI of the World Health Organization, which is adopted by the American College of Obstetrics for pregnant patients. Body Mass Index was determined with BMI=weight (kg) height^-2^ (cm) formula. Patients were assigned into Group 1 (n=20) and Group 2 (n=20), according to BMI being lower and higher than 30 kg m^-2^, respectively.

Every patient taken to the operation room was monitored non-invasively for arterial pressure (mmHg), heart rate (HR per minute), and peripheral oxygen saturation (SpO_2_). Patients were informed about the operation, and all patients had USG by the same practitioner. All ultrasonographic investigations were performed with USG (SDU-450 XL, Kyoto, Japan).

Each patient’s head was placed into mild extension in the supine position. The patient was ordered to breathe slowly in order to avoid airway changes due to breathing. Ultrasonographic measurement was performed from the mid-front part of the neck using a linear probe (5-10 mHz). Ultrasonographic procedure started from the level of true vocal cords, and tracheal arc was monitored by moving the probe slowly towards the caudal direction in order to avoid disorder between the tracheal cartilage and tracheal ring. The diameter of the tracheal transverse air shadow in the subglottic area was measured three times and mean value was calculated.^12^ Besides patients’ demographic data, and modified Mallampati score^13^ (Table-I) were evaluated. Thyromental distance and sternomental distance measurements were made and recorded. Patients were preoxygenated with 100% oxygen for 3 minutes. Anesthesia was standardized in all patients using a rapid-sequence induction protocol. Patients were given propopol 2 mg/kg, and succinylcholine 1.5 mg/kg, or rocuronium 0.6 mg/kg. After cricoid pressure was applied and the trachea was intubated anesthesia was maintained with sevoflurane in a mixture of nitrous oxide 2 L/min and oxygen 2 L/min, and the lungs of all patients were mechanically ventilated to maintain an ETCO_2_ concentration of 32–37 mmHg. Patients underwent laryngoscopy by the same advanced specialist anesthesiologist after general anesthesia application and Cormack-Lehane score^14^ (Table-II) was recorded. After delivery, midazolam 2 mg and fentanyl 100 µg were given intravenously.

The sample sizes were calculated with the assumption of a possible in ultrasonic measurements at least of 20% between any two groups. Therefore 20 patients were assigned into each group in order to obtain an alpha error of 5% and statistical power of 80%.

***Statistical Analysis: ***Statistical analysis in this study were made using NCSS (Number Cruncher Statistical System) 2007 Statistical software (Utah, USA). In addition to definitive statistical methods (mean standard value, median, interquartile range), the Mann-Whitney U test was used to compare groups, and the chi-square test was used to compare qualitative data. A p<0.05 was accepted as statistically significant.

## RESULTS

There were no statistically significant differences between groups regarding mean age range (p=0.694) and mean number of pregnancy range (p=0.628). Group 2 had statistically significant higher mean values in terms of body weight (p=0.0001), pre-pregnancy body weight (p=0.0001), and BMI (p=0.0001) compared to Group 1 (Table-III). There were no statistically significant differences between groups regarding ASA scores (p=0.292), comorbid disease presence range (p=0.376) (Table-IV). There were no statistically significant differences between groups regarding mean values of the modified Mallampati scores (p=0.068) and Cormack-Lehane scores (p=0.365) (Table-V). There were no statistically significant differences between groups regarding the measurements of thyromental distance (p=0.539), sternomental distance (p=0.535), and ultrasonic measurement values (p=0.160) (Table-VI).

## DISCUSSION

Obesity is defined as having a BMI higher than 30 kg m^-2^. Due to massive adipose tissue presence, oral opening and jaw movement limitations and tightening in pharyngeal distance are seen in obese patients.^15^ Endotracheal intubation is more difficult in obese patients and pregnant women in comparison to patients with normal body weight.^8^^,^^16^^,^^17^ It is reported that difficult intubation risk increases with a rise in BMI, particularly in those with a BMI higher than 35 kg m^-2^, where in the incidence of difficult intubation is as high as 15%.^8^^,^^18^ Difficult or failed intubation in pregnant women causes an increase in maternal morbidity and mortality by increasing the risk for hypoxic cardiac arrest and/or pulmonary aspiration. Therefore, careful examination must be carried out prior to anesthesia to determine the predictive factors for difficult intubation.^3^ There are no specific tests to evaluate the airway in obstetric and obese patients, and classical tests are used for these populations. However, none of these methods are adequate for predicting difficulty in endotracheal intubation.^19^ Short sternomental distance and thyromental distance, limitations in head-neck and jaw movements, and modified Mallampati scores higher than 3 indicate a high possibility of difficult intubation.^8^ Although difficult intubation risk is reported to be 34% after a positive Mallampati test, this finding cannot be used as a predictive parameter by itself.^17^ Our study showed no statistically significant difference between groups in the clinical tests. None of our patients had a modified Mallampati score or Cormack-Lehane score of 4. Two patients in the group with BMI>30 kg m^-2 ^had difficult intubation. Both of these patients had a modified Mallampati score of 2 and a Cormack-Lehane score of 3. While the first patient was intubated on second attempt with blind intubation technique, the latter was intubated on the third attempt without usage of any alternative device. We have not expected any ventilation difficulty in our patients.

**Table-I:** Modified Mallampati Score

Class 1: Tonsils, uvula and soft palate are fully visible.
Class 2: Soft palate and uvula are visible.
Class 3: Soft palate and base of uvula are visible.
Class 4: Only hard palate is visible.

**Table-II:** Cormack-Lehane Scores

1. Grade: Glottis is fully visible.
2. Grade: Glottis is partially visible.
3. Grade: Only epiglottis is seen.
4. Grade: Neither the epiglottis nor glottis seen.

**Table-III:** Mean age, body weight, body weight before pregnancy, number of pregnancy and BMI in groups

	*Group 1* *(n=20)*	*Group 2* *(n=20)*	*p*
Age (year)	29,15±5,74	28,25±6,14	0.694
Body weight (kg)	68,2±7,61	85,75±10,11	0.0001*
Body weight before pregnancy (kg)	58,15±6,11	71,25±11,31	0.0001*
Number of pregnancy	2,65±0,81	2,60±1,27	0.628
BMI	26,49±2,63	32,84±2,42	0.0001*

*p<0.001 (Mean±SD), BMI: Body Mass Index

**Table-IV:** ASA score and co-morbid diseases in groups

		*Group 1* *(n=20)*		*Group 2* *(n=20)*		*P*
ASA Score	1	19	95,00%	17	85,00%	p=0.292
	2	1	5,00%	3	15,00%	
Co-morbid Diseases	No	18	90,00%	16	80,00%	p=0.376
	Yes	2	10,00%	4	20,00%	

*p<0.05 (Mean±SD), ASA: American Society of Anesthesiologists

**Table-V:** Modified Mallampati Score and Cormack-Lehane Score in groups

		*Group 1* *(n=20)*		*Group 2* *(n=20)*		*p*
Modified Mallampati Score	1	13	65,00%	6	30,00%	p=0.068
	2	7	35,00%	13	65,00%	
	3	0	0,00%	1	5,00%	
Cormack-Lehane Score	1	13	65,00%	9	45,00%	p=0.365
	2	6	30,00%	8	40,00%	
	3	1	5,00%	3	15,00%	

*p<0.05

**Table-VI:** Thyromental distance, sternomental distance and ultrasonographic measurement in groups

	*Group 1* *(n=20)*	*Group 2* *(n=20)*	*p*
Thyromental distance (cm)	7,28±1,28	7,58±0,75	0.539
Sternomental distance (cm)	11,25±1,73	11,55±1,77	0.535
Ultrasonographic measurement (mm)	16,78±2,13	17,69±1,91	0.160

*p<0.05 (Mean±SD)

Although using USG findings in suggesting difficult intubation can be useful, the data is limited.^6^ In various clinical trials, USG-related difficult intubation criteria were investigated but results are controversial.^6^^,^^17^^,^^20^^-^^22^ Kundra et al^6^ reported pretracheal soft tissue measurements via USG at vocal cord level in obese patients as a good predictor for difficult intubation. However Komatsu et al^17^ reported that pretracheal soft tissue thickness is not an indicator of difficult laryngoscopy in morbidly obese patients. Adhikari et al^20^ measured the soft tissue of the neck and tongue thickness via USG in normal patients, and found no statistically significant difference between measurements and clinical scan tests. Another study found that pretracheal soft tissue thickness is not a good indicator for difficult intubation in morbidly obese patients.^21^ Wojtczak’s study^22^ showed that hyomental space measurements using USG can be useful in predicting difficult intubation. One of the anatomical changes pregnancy causes is pharyngolaryngeal tightening, which is worsened by obesity. Ultrasonography is defined as a reliable tool for determining the narrowest diameter in the cricoid lumene. In their study, Prasad et al^23^ compared USG and anatomic airway parameters using USG and computerized tomography (CT). Results showed that all structures able to be evaluated via CT can be monitored using USG. Lakhal et al^12^ compared the diameter of the cricoid lumene measurements using USG and magnetic resonance imaging in healthy, young adults, and concluded that diameter of the upper airway can be reliably measured using USG. Our study concluded that ultrasonic measurements of the subglottic area does not change according to BMI. We did not find any differences between conventional preoperative airway evaluation tests and USG findings in obese pregnant patients. 

In conclusion, ultrasonographic evaluation of the upper airway may provide anatomic information, but it is not useful alone. We thought that ultrasonic measurement could not give us valuable information in obese or non-obese pregnant women. We suggest that obesity in pregnant women does not cause a significant change in thyromental distance, sternomental distance, modified Mallampati score, or Cormack-Lehane score in ultrasonographic measurements although USG is a reliable, non-invasive airway measurement, and it is safe to use during pregnancy. However, further clinical experience and studies are required to assess this conclusion in greater depth.

## Authors Contribution:

MTA: Concept, Design, Supervision, Funding-Materials, Analysis and/or Interpretation of data and preparing the manscript. KE: Design, Supervision, Funding-Materials, Analysis and/or Interpretation of data.SNS: Supervision, Data Collection and/or Processing. TAU: Funding-Materials, Literature search. GOU: Funding-Materials, Data Collection and/or Processing and Literature search. AA: Critical Review, Analysis and/or Interpretation of data.

## Notes

***Funding Support:*** No.
